# Plasma membrane Ca^2+^‐ATPase 1 is required for maintaining atrial Ca^2+^ homeostasis and electrophysiological stability in the mouse

**DOI:** 10.1113/JP274110

**Published:** 2017-11-09

**Authors:** Yanwen Wang, Claire Wilson, Elizabeth J. Cartwright, Ming Lei

**Affiliations:** ^1^ Department of Pharmacology University of Oxford Oxford UK; ^2^ Division of Cardiovascular Sciences, Faculty of Biology, Medicine and Health, Manchester Academic Health Science Centre University of Manchester Manchester UK

**Keywords:** plasma membrane Ca^2+^ ATPase 1 (PMCA1), heart, atrial myocyte Ca^2+^ homeostasis

## Abstract

**Key points:**

- The role of plasma membrane Ca^2+^‐ATPase 1 (PMCA1) in Ca^2+^ homeostasis and electrical stability in atrial tissue has been investigated at both organ and cellular levels in mice with cardiomyocyte‐specific deletion of PMCA1 (PMCA1^cko^)
- The PMCA1^cko^ hearts became more susceptible to atrial arrhythmic stress conditions than PMCA1^loxP/loxP^ hearts.
- PMCA1 deficiency alters cellular Ca^2+^ homeostasis under both baseline and stress conditions.
- PMCA1 is required for maintaining cellular Ca^2+^ homeostasis and electrical stability in murine atria under stress conditions.

**Abstract:**

To determine the role of plasma membrane Ca^2+^‐ATPase 1 (PMCA1) in maintaining Ca^2+^ homeostasis and electrical stability in the atrium under physiological and stress conditions, mice with a cardiomyocyte‐specific deletion of PMCA1 (PMCA1^cko^) and their control littermates (PMCA1^loxP/loxP^) were studied at the organ and cellular levels. At the organ level, the PMCA1^cko^ hearts became more susceptible to atrial arrhythmias under rapid programmed electrical stimulation compared with the PMCA1^loxP/loxP^ hearts, and such arrhythmic events became more severe under Ca^2+^ overload conditions. At the cellular level, the occurrence of irregular‐type action potentials of PMCA1^cko^ atrial myocytes increased significantly under Ca^2+^ overload conditions and/or at higher frequency of stimulation. The decay of Na^+^/Ca^2+^ exchanger current that followed a stimulation protocol was significantly prolonged in PMCA1^cko^ atrial myocytes under basal conditions, with Ca^2+^ overload leading to even greater prolongation. In conclusion, PMCA1 is required for maintaining Ca^2+^ homeostasis and electrical stability in the atrium. This is particularly critical during fast removal of Ca^2+^ from the cytosol, which is required under stress conditions.

## Key points

- The role of plasma membrane Ca^2+^‐ATPase 1 (PMCA1) in Ca^2+^ homeostasis and electrical stability in atrial tissue has been investigated at both organ and cellular levels in mice with cardiomyocyte‐specific deletion of PMCA1 (PMCA1^cko^)
- The PMCA1^cko^ hearts became more susceptible to atrial arrhythmic stress conditions than PMCA1^loxP/loxP^ hearts.
- PMCA1 deficiency alters cellular Ca^2+^ homeostasis under both baseline and stress conditions.
- PMCA1 is required for maintaining cellular Ca^2+^ homeostasis and electrical stability in murine atria under stress conditions.

**Abbreviations list**
AF: atrial fibrillation
AP: action potential
APD_90_: action potential duration at 90%
AT: atrial tachycardia
BCL: basal cycle length
DAD: delayed after‐depolarization
EAD: early after‐depolarization
ERP: effective refractory period
HR: heart rate
LTCC: L‐type Ca^2+^ channel
MAP: monophasic action potential
NCX: Na^+^/Ca^2+^ exchanger
PES: programmed electrical stimulation
PMCA: plasma membrane Ca^2+^‐ATPase
RyR: ryanodine receptor
SR: sarcoplasmic reticulum

## Introduction

As one of the most crucial ions in the cell, Ca^2+^ is involved in cardiac electrophysiological activity, excitation–contraction coupling, contractile function, energy balance, cell death and gene transcription. During each action potential (AP) of cardiac myocytes, Ca^2+^ fluxes into the cell through voltage gated L‐type Ca^2+^ channels (LTCCs), which are activated by AP‐induced membrane depolarization. The primary role of this Ca^2+^ influx is to trigger sarcoplasmic reticulum (SR) Ca^2+^ release, through the process of Ca^2+^‐induced Ca^2+^ release. The Ca^2+^ released from the SR Ca^2+^ store then causes myocytes to contract. When contraction ends the Ca^2+^ ions are taken up by SERCA2a back into the SR, with the remainder being extruded by two outward transport mechanisms in the sarcolemma that are responsible for calcium extrusion: (i) the plasma‐membrane Ca^2+^‐ATPase (PMCA), which ejects calcium to the extracellular compartment using energy derived from ATP hydrolysis and is dependent on calmodulin (Bers, 2002), and (ii) the Na^+^/Ca^2+^ exchanger (NCX), which counter‐transports 1 molecule of calcium in exchange for 3 molecules of sodium utilizing the sodium gradient across the plasma membrane as an energy source (Blaustein & Lederer, 1999). In cardiomyocytes, the trans‐sarcolemmal calcium ejection is performed mostly by NCX. Accumulating evidence suggests that NCX transports approximately 10–15 times more calcium (depending on species) than PMCA (Bers, 2000, 2002).

The quantitative contribution of the SR, NCX and PMCA to Ca^2+^ removal is species‐dependent. In rabbit ventricular myocytes, the SR is responsible for approximate 70% of the Ca^2+^ uptake and NCX removes about 28%. It is believed therefore that PMCA in cardiomyocytes has only a limited role in calcium transport, and makes a minor contribution to the excitation–contraction coupling process (Oceandy *et al*. 2007). PMCA and the mitochondrial Ca^2+^ uniporter contribute 1% each (Bassani *et al*. 1994). In rat, about 92% of the Ca^2+^ is taken up by the SR; only 7% is extruded by NCX and 1% by PMCA and mitochondria (Bers, 2002). In atrial myocytes, the Ca^2+^ cycling is slightly different from ventricular myocytes. In rat and mouse, atrial myocytes lack t‐tubules, but contain a form of transversely oriented tubules formed by internal SR membrane, described as Z‐tubules (Yamasaki *et al*. 1997). According to results from Bootman and colleagues, the localization pattern of the ryanodine receptor (RyR) in atrial myocytes has some similarity to that observed in ventricular myocytes. Due to the lack of t‐tubules, the localization of NCX is only predominant on the circumference of the atrial myocytes. Therefore excitation–contraction coupling in atrial myocytes initially occurs at the cell periphery (Bootman *et al*. 2006).

Four isoforms of the PMCA pump (PMCA1–4) encoded by separate genes have been identified in mammals. Among them, PMCA1 is predominantly expressed in cardiac tissues. It has been suggested that PMCA1 pumps only make a minor contribution to Ca^2+^ extrusion compared with NCX and to date little is known about the physiological role of PMCA1 in atrial myocytes.

As the global deletion of PMCA1 is embryonic lethal, we used the Cre‐loxP system to disrupt *PMCA1* gene expression specifically in cardiac tissue to generate a mouse cardiomyocyte‐specific knockout model of PMCA1 (PMCA1^cko^). Using this unique model we clarified the role of PMCA1 in maintaining atrial Ca^2+^ homeostasis and electrophysiological stability in the atria. The study was carried out in several steps. Firstly, the expression of PMCA1 in the atrial tissue was studied by immunostaining tissue sections, and the deletion efficiency of PMCA1 in the atrium of PMCA1^cko^ mice was examined. Then, the *in vivo* and *ex vivo* electrophysiological cardiac phenotypes of PMCA1^cko^ mice and their control littermates, PMCA1^loxP/loxP^ mice, were investigated by ECG and monophasic action potential (MAP) recordings under 1 mm [Ca^2+^]_o_ and 2 mm [Ca^2+^]_o_ (Ca^2+^ overload) conditions with and without programmed electrical stimulation (PES). Finally a series of single cell studies were conducted on atrial myocytes isolated from PMCA1^cko^ and PMCA1^loxP/loxP^ mice using patch clamping, epifluorescence Ca^2+^ measurements and confocal Ca^2+^ imaging.

## Methods

### Ethical approval

All animal experiments were performed on adult mice (16–20 weeks old) in accordance with the UK Animals (Scientific Procedures) Act 1986, were approved by the University of Manchester ethical committee and were conducted in accordance with the national guidelines under which the institution operates. All mice used in this study were maintained in a pathogen‐free facility at the University of Manchester. The authors confirm that they have taken all steps to minimize the animals’ pain and suffering. Our work complies with *The Journal*’s policy and regulations (Grundy, 2015).

### Animals

Cardiomyocyte‐specific PMCA1 knockout mice were generated using Cre–*loxP* technology. PMCA1 floxed mice in which *loxP* sites were inserted to flank exon 2, containing the ATG start codon of the *PMCA1* gene, were generated as described in Ryan *et al*. (2015). PMCA1 floxed mice were crossed with αMHC‐Cre transgenic mice in which Cre is driven by the α‐myosin heavy chain promoter to induce recombination events in atrial and ventricular cardiomyocytes (Agah *et al*. 1997). All protocols involving the husbandry and uses of animals were licensed by the UK Home Office.

Adult (3–4 months old) male PMCA1^cko^ mice and their control littermates PMCA1^loxP/loxP^ were used in this study. Generation of PMCA1^loxP/loxP^ mice was previously described in detail by Ryan *et al*. (2015). PMCA1^cko^ mice are generated by crossing the PMCA1^loxP/loxP^ mice with αMHC‐Cre mice (Agah *et al*. 1997) (kindly provided by M. D. Schneider, National Heart and Lung Institute, Imperial College London), which express Cre recombinase in atrial and ventricular cardiomyocytes.

For atrial single cell isolation, in order to prevent blood clot formation, animals were injected with 0.2 ml heparin (1000 U ml^−1^) under general anaesthetic for 5 min by injecting the anaesthetic agent Avertin (2,2,2‐tribromoethanol, 300 mg kg^−1^ body weight in 0.3–0.4 ml injecting saline solution, i.p.) before Schedule 1 killing. Animals were killed by cervical dislocation, and the heart was removed immediately and placed in ice‐cold Krebs–Ringer solution (concentration (mm): 120 NaCl, 4 KCl, 1.3 MgSO_4_·7H_2_O, 1.2 NaH_2_PO_4_·2H_2_O, 25.2 NaHCO_3_, 5.8 glucose, oxygenated with 95% oxygen and 5% carbon dioxide) during transport for *ex vivo*, single cell and molecular studies.

Animals for *in vivo* studies were anaesthetized by inhalation of 1.5–2% isoflurane mixed with 100% oxygen at flow rate 1∼1.5 l min^−1^, and kept warm on a heating pad.

### *Ex vivo* ECG and MAP recordings

Animals were killed by cervical dislocation and the heart was quickly removed, cannulated and perfused via a Langendorff system. The heart was perfused with Krebs–Ringer solution with a constant flow rate of 4 ml min^−1^ at 37°C to allow good circulation of the coronary artery system. After cannulation of the aorta, a metal clip was used to clamp the aorta to the cannula. The excess tissues and lungs were removed, and a thread was used to tie the aorta just below the clip to maintain constant pressure to the heart. To obtain stabilized and reliable recordings, the heart was left on the Langendorff system for 15 min to allow it to reach a steady state before recording started. The two ECG electrodes were placed opposite each other on the left atrium and ventricle apex; the MAP electrode was placed on the right atrium. ECG and MAP recordings were performed simultaneously. The positions of the electrodes were optimized to be consistent between different hearts. Noise was minimized by grounding the devices and set‐ups to the earth. The ECG and MAP recordings were made by LabChart 5 software (ADInstruments, Oxford, UK). The onset of the QT interval was taken to correspond either to the Q wave deflection or, if this was absent, the base of the R wave. The end of the QT interval was taken as the time point after the T wave peak when the first derivative of the voltage trace (d*V*/d*t*) became zero. When undershoots were present, the end of the QT interval was estimated from the minimum value of the undershoot. The QT interval was also corrected for heart rate (HR) using the following formula: QT_c_ = QT/(RR/100)^1/2^, where QT_c_ is the corrected QT interval and RR is the R–R interval (Mitchell *et al*. 1998).

### Programmed electrical stimulation

A pair of bipolar pacing electrodes (custom‐made) were placed on the atrial septum and the PES, controlled by Spike2 software (Cambridge Electronic Design, Cambridge, UK) was applied. Propensity to atrial arrhythmias in Langendorff‐perfused hearts was assessed by PES. Pacing trains of eight stimuli (S1) delivered at a basic cycle length of 100 ms were followed by a single premature extra stimulus (S2) introduced at S1–S2 intervals progressively shortened with successive stimulus cycles repeated until arrhythmia was induced or the atrial refractory period was reached. A burst‐pacing protocol applied atrial pacing in trains of 20 S1 stimuli at progressively shorter intervals from an initial cycle length of 100 ms until an arrhythmia was induced or a cycle length of 30 ms was reached. Arrhythmia was defined as three or more consecutive premature atrial waveforms. Tachycardia showing regular waveforms was defined as atrial tachycardia (AT), whereas irregular fibrillating waveforms were considered to represent atrial fibrillation (AF).

### Single atrial myocyte electrophysiology and [Ca^2+^]_i_ measurements

#### Cell isolation

Mice were killed by a Schedule 1 procedure. The heart was removed and then immediately cannulated and Langendorff‐perfused (with (mm): 115 NaCl, 10 Hepes‐acid, 11.1 glucose, 1.2 NaH_2_PO_4_, 1.2 MgSO_4_, 4 KCl, 50 taurine, pH 7.34 with NaOH) to flush away any remaining blood. Then the heart was perfused with the enzyme solution, which contained 0.5 mg ml^−1^ collagenase Type‐2 (40E11903 CLS‐2, Worthington Co., Lakewood, NJ, USA, 280 u mg^−1^, 395 u mg^−1^ caseinase, 4.7 u mg^−1^ clostripain, 0.4 u mg^−1^ Tryptic activity) and 0.05 mg ml^−1^ protease (Sigma‐Aldrich, Gillingham, UK) dissolved in isolation solution (mm: 134 NaCl, 10 Hepes acid, 11.1 glucose, 1.2 NaH_2_PO_4_, 1.2 MgSO_4_, 4 KCl, pH 7.34 with NaOH) for approximately 5–6 min. When the heart became flaccid, the ventricles were chopped up into small pieces and washed in taurine solution to prevent over‐digestion by the remaining enzyme solution; myocytes were isolated from ventricular tissue by gently suspending, and further digestion was applied when the tissue was under‐digested.

The atrial myocytes were isolated by a two‐step procedure. After the heart was perfused with the first enzyme for approximately 5–6 min, the left and right atria were dissected from the heart and incubated in second enzyme solution containing 3 mg ml^−1^ collagenase type‐2, 0.3 mg ml^−1^ protease and 0.3 mg ml^−1^ elastase (Serva, elastase from porcine pancreases) dissolved in isolation solution in a 37°C water bath for another approximately 5 min. When the atrium became soft and flabby, the tissue was gently torn up and suspended allowing isolation of atrial myocytes. Further incubations were applied as necessary.

The isolated cardiomyocytes were washed in 0.1% (w/v) BSA in taurine solution to remove any excessive enzyme and stop further digestion, and stored in normal Tyrode solution (concentration (mm): 134 NaCl, 4 KCl, 1 CaCl_2_, 1.2 MgCl_2_, 11.1 glucose, 10 Hepes, pH 7.4 with NaOH) at room temperature.

### Loading Ca^2+^ indicators

#### Indo‐1

Each 50 μg of Indo‐1 (acetoxymethyl (AM) ester form, cell permeant, I‐1223, Thermo Fisher Scientific, Paisley, UK) powder was dissolved in 50 μl 25% Pluronic acid (Sigma‐Aldrich) in dimethyl sulfoxide (DMSO; 5 × 5 ml; D2650, Sigma‐Aldrich); 1 mg of Pluronic acid powder was dissolved in 4 μl DMSO to make 25% (w/v); vortexing with heating was applied during the process of dissolving. The 25% Pluronic acid DMSO stock solution was used to dissolve the Ca^2+^ indicator powder. Atrial myocytes were isolated as described above and loaded with the membrane permeable AM form of the fluorescent Ca^2+^ indicator for 5 min incubation in 37°C water bath, and then the supernatant was changed to Tyrode solution with 1 mm [Ca^2+^]_o_ concentration for 45 min to allow de‐esterification. The myocytes were placed in the chamber on the microscope and left for 5∼10 min for stabilization before starting the perfusion with Tyrode solution at room temperature in the dark. The cells with poor quality were washed away and the healthy cells were selected for experiments. Once a single cell was chosen, the microscope shutter was adjusted to ensure the field was as small as possible and the cell just fitted in. The Ca^2+^ transients detected by Indo‐1 were presented as the ratio of *F*_405_/*F*_490_ after subtracting the background fluorescence for the cell in the field of view. Ca^2+^ measurements based on the Indo‐1 experiments were calculated based on ratios of *F*_405_/*F*_490_.

#### Fluo‐4

Cells were loaded with the AM form of the Ca^2+^ indicator Fluo‐4, Fluo4‐AM (Molecular Probes, Thermo Fisher Scientific, F‐14201) to provide a wide range of sensitivity to changes of [Ca^2+^]_i_. Fluo‐4 powder was dissolved in DMSO containing 25% Pluronic acid (Sigma‐Aldrich) at a concentration of 1 μg μl^−1^. Myocytes were then incubated with Fluo‐4 Ca^2+^ indicator solution for 15 min followed by 45 min in normal Tyrode solution to permit the Ca^2+^ indicator to de‐esterify. Measured fluorescence was normalized to resting levels (*F*/*F*_0_).

#### Voltage clamping

Patch electrodes were pulled from borosilicate glass (1.5 mm o.d. × 1.17 mm i.d., CEI, Harvard Apparatus, Cambridge, UK) on a gravity vertical puller (Model PP‐830, Narishige, Japan). The fire‐polished electrodes had a resistance of 2.5–3 MΩ when filled with patch electrode internal solutions, which contained (in mm): 120 CsCl, 12 NaCl, 10 Hepes, 20 tetraethyl ammonium chloride, 0.1 EGTA, 5 MgCl_2_ and 5 K_2_ATP, titrated to pH 7.2 with CsOH. The cells were continuously perfused with normal Tyrode solution, which contained (in mm) 134 NaCl, 11.1 glucose, 1 CaCl_2_, 10 Hepes, 1.2 MgCl_2_, 4 KCl, 5 4‐aminopyridine, 0.1 BaCl_2_, titrated to pH 7.4 with NaOH. 4‐Aminopyridine and BaCl_2_ were used to block the potassium current. Experiments were performed at room temperature (20–22°C). Whole cell membrane current and membrane voltage were recorded using an Axopatch 200B Amplifier (Molecular Devices, Sunnyvale, CA, USA) and data requisition and analysis performed by the pCLAMP 10 programme (Molecular Devices). Cells were stimulated with depolarizing pulses (100 ms duration from −40 to 0 mV) at different stimulating frequencies.

#### [Ca^2+^]_i_ measurements

Fluorescence was excited at a wavelength of 488 nm and emitted at a wavelength of 494/506 nm – conversion of [Ca^2+^]_i_ assumed a *K*_d_ of 345 nm. The rates of decay of the systolic and caffeine‐evoked Ca^2+^ transients were obtained by fitting single exponential functions to the decay phase of the transient. In each cell the decay curve was fitted over the same range of [Ca^2+^]_i_.

#### Calculation of Ca^2+^ fluxes

Ca^2+^ fluxes were calculated by integration of membrane currents. Ca^2+^ influx through the LTCCs was calculated by integrating the L‐type Ca^2+^ current (*I*_LTCC_) and Ca^2+^ efflux associated with the Ca^2+^ transient by integration of NCX tail current (*I*_NCX_) immediately after repolarization. For these calculations, the current value corresponding to the minimum [Ca^2+^]_i_ reached after systole was used as the baseline current level.

### SR Ca^2+^ content measurements

SR Ca^2+^ content was assessed following trains of action potentials by rapidly switching to voltage clamp control at a holding potential of −80 mV and applying 10 mm caffeine to deplete the SR Ca^2+^ store. The resultant NCX current was integrated and corrected for Ca^2+^ removal by non‐electrogenic mechanisms.

### Spinning disk confocal microscopy

Mouse atrial myocytes were isolated and loaded with the fluorescent Ca^2+^ indicator Fluo‐4 (Thermo Fisher Scientific) at a final concentration of 3 μm. The Ca^2+^ spark images were recorded using a Nikon Aviovert 200 inverted microscope with an Andor iQ confocal system attached with a Nipkow spinning disk confocal set‐up (CSU‐10, Andor Technology, Belfast, UK). Laser excitation of Fluo‐4 was at 488 nm by diode laser (Cairn Research Ltd, Faversham, UK) and the emitted light was collected using a 515 nm long pass filter using an Andor iXON897 EM‐CCD camera (Andor Technology) at a frame rate of 50 Hz. Ca^2+^ spark images were recorded and analysed using Andor iQ software.

### Linescan confocal microscopy

The linescan images of mouse atrial myocytes were recorded with the Leica DMIRB inverted confocal microscope system attached with a Leica TCS NT scanning head under line scan mode at a rate of 2.6 ms per line. Myocytes were visualized with a ×100 oil immersion objective lens. Excitation light of 488 nm was provided by an argon ion laser system (Uniphase, Milpitas, CA, USA) and emitted light was collected at 515 nm wavelength by using a long pass filter. The images were recorded using Leica TCS NT software and analysed using ImageJ software.

### Immunohistochemistry

The atrium tissue was cut off immediately after killing the mice, and was embedded and frozen completely in Optimal Cutting Temperature compound (VWR International Ltd, Lutterworth, UK) by liquid nitrogen and isopentane (Sigma‐Aldrich). The frozen tissue was sliced by the Leica CM 3050S cryostat (Leica Microsystems (UK) Ltd) on Superfrost Plus histological slides (VWR International Ltd). Slides were incubated with 4% formaldehyde (Sigma‐Aldrich) at room temperature for 30 min followed by three washes of 10 min each with PBS (Sigma‐Aldrich), then permeablized with 0.1% Triton X‐100 (Sigma‐Aldrich) for 30 min. After three washes of 10 min each wash with PBS the sections were incubated with 3% (w/v) BSA in PBS at room temperature for 30 min. The targeting proteins were labelled by primary antibody diluted in 3% (w/v) BSA in PBS at 4°C overnight, and then washed three times of 10 min each with PBS. The primary antibody was then tagged with corresponding secondary antibody, either fluorescein isothiocyanate or Cy3 dye diluted in 3% (w/v) BSA in PBS at room temperature for 2.5 h in a dark room. The tissues section was then washed and mounted with Vectashield mounting medium (H‐1000, Vector Laboratories Ltd, Peterborough, UK) and sealed with nail varnish.

### Western blots

Protein from atrial tissue was extracted in Triton lysis buffer (mm: 20 Tris–HCL, pH 7.4, 137 NaCl, 2 EDTA, 25 β‐glycerophosphate, 1 orthovanadate, 1 phenylsulphfonyl fluoride, 1% Triton X‐100, 10% glycerol, 10 μg ml^−1^ leupeptin, 10 μg ml^−1^ aprotinin). Immunoblot analyses of protein extracts (50 μg) with antibody against PMCA1 (1:1000, Abcam, Cambridge, UK) and α‐tubulin (1:1000, Merck Chemicals Ltd) were repeated three times on three individual animals. Immunocomplexes were detected by enhanced chemiluminescence with the corresponding secondary antibody; anti‐rabbit IgG horseradish peroxidase (1:2000, Jackson ImmunoResearch Laboratories, West Grove, PA, USA), or anti‐goat IgG coupled to horseradish peroxidase (1:2000, Merck Chemicals Ltd).

### RNA isolation

RNA was extracted from ventricle samples using the Trizol method (Thermo Fisher Scientific). Due to the small size of atria (≤30 mg), RNA was isolated from atrial samples using the RNeasy fibrous tissue mini kit (Qiagen, Manchester, UK), following the manufacturer's protocol. RNA concentration was quantified using a NanoDrop 1000 spectrophotometer (Thermo Fisher Scientific) and RNA (1 μg μl^−1^) was reverse transcribed into cDNA using the High Capacity RNA‐to‐cDNA kit (Thermo Fisher Scientific).

### Quantitative PCR

Gene expression was assessed using qPCR. TaqMan primers and probes were used to measure mRNA expression levels of PMCA4 in the atria and ventricle of PMCA1^cko^ and PMCA1^loxP/loxP^ mice, and GAPDH was used as the housekeeping gene. qRT‐PCR experiments were prepared using TaqMan® Gene Expression Master Mix and TaqMan® Gene Expression Assays (Thermo Fisher Scientific) according to manufacturer's instructions.

### Isoprenaline mini‐pump

Osmotic mini‐pumps (ALZET model 1007D1, ALZET Osmotic Pumps, Cupertino, CA, USA) were implanted subcutaneously into mice. Isoprenaline (Sigma‐Aldrich) was dissolved in 0.9 % NaCl at a concentration calculated to deliver 10 μg g^−1^ body weight day^−1^ for up to 7 days at an infusion rate of 0.25 μl h^−1^. Pumps were filled with either isoprenaline solution or 0.9% NaCl (vehicle control) before being implanted in mice under anaesthesia (isoflurane). The analgesic buprenorphine (0.1 mg kg^−1^ body weight) was injected subcutaneously immediately prior to surgery. Post‐surgery the mice were continuously monitored until awake and were then transferred to a 37°C warming cabinet for 2 h. The mice continued to be regularly monitored in the days post‐surgery. *In vivo* ECG recordings were measured before and 7 days following isoprenaline stimulation.

### Dobutamine

Dobutamine hydrochloride (Sigma‐Aldrich) was diluted in 0.09% NaCl to a concentration of 0.1 μg μl^−1^. Mice received a single intraperitoneal injection of dobutamine at a dose of 1.5 ug g^−1^ body weight or an equivalent volume 0.9% NaCl (vehicle). *In vivo* ECG recordings were measured before and periodically throughout the experiment until peak heart rate response was achieved and the heart rate began to return to normal.

### *In vivo* electrocardiography

*In vivo* ECG was carried out on 3‐month‐old mice. Recording were conducted under general anaesthetic induced by isoflurane. Subcutaneous electrodes were inserted and three‐lead ECG recordings (Power Lab/4SP, ADInstruments) were made. LabChart Pro (ADInstruments) was used to pace and analyse *in vivo* ECG recordings. Heart rate and the following intervals were measured: RR, PR, QRS, QT and JT. Corrected QT duration for heart rate (QTc) was calculated using the Bazett formula (QTc = QT/√(60/HR)). The mice were killed by cervical dislocation after the ECG recording was completed and the hearts were harvested for molecular or immunohistological studies.

### Data analysis and statistical methods

**Figure 1:**
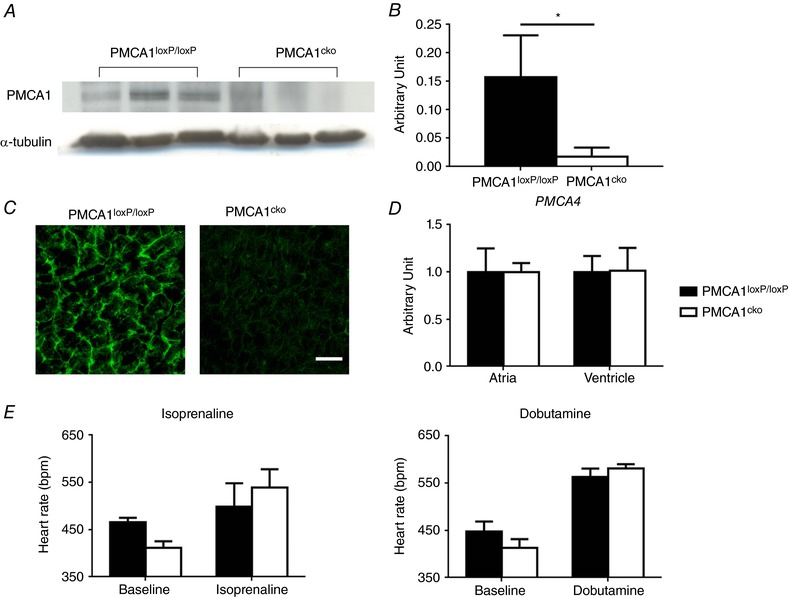
Expression of PMCA1 is significantly reduced in the atria of PMCA1^cko^ mice compared to PMCA1^loxP/loxP^ controls *A*, a representative western blot image of PMCA1 protein expression in atrial extracts. α‐Tubulin was used as a loading control. *B*, the level of expression was quantified showing a significant reduction in PMCA1/α‐tubulin ratio in PMCA1^cko^ mice atria (0.158 ± 0.073/0.017 ± 0.016, *P* = 0.026, 3 replicates from 3 animals each). *C*, the efficiency of the PMCA1 knockout in PMCA1^cko^ mouse atria was further confirmed by immunohistochemical staining of atrial sections compared with PMCA1^loxP/loxP^ mice. *D*, the mRNA levels of PMCA4 in both atria and ventricle remained unchanged in PMCA1^loxP/loxP^ and PMCA1^cko^ mice. *E*, the *in vivo* heart rates of PMCA1^loxP/loxP^ and PMCA1^cko^ mice showed no significant difference with either 1‐week isoprenaline treatment or acute dobutamine injection treatment. The bars show the mean + SD.

**Figure 2:**
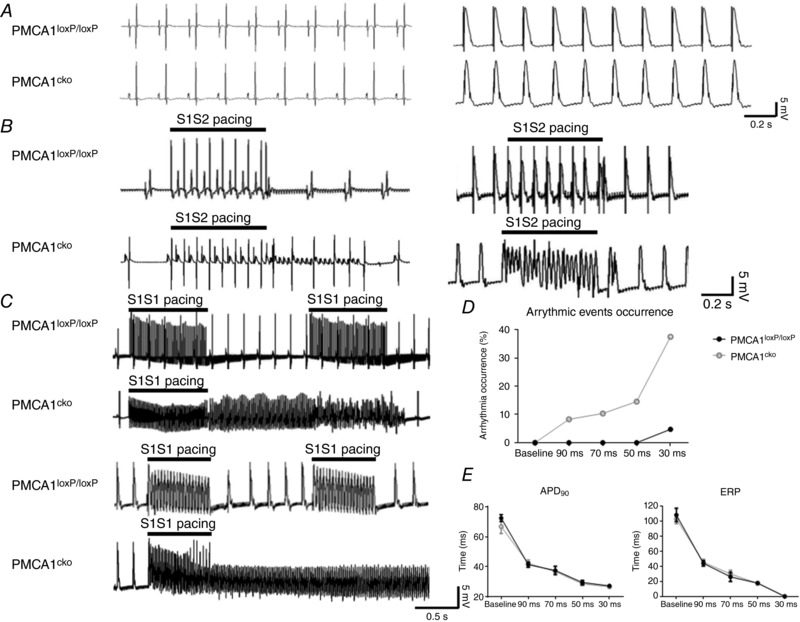
*Ex vivo* Langendorff recordings of ECG and atrial MAP of PMCA1^cko^ and PMCA1^loxP/loxP^ hearts under baseline condition at 1 mm [Ca^2+^]_o_ *A*, representative traces of the *ex vivo* Langendorff ECG (left) and MAP (right) recordings. There was no significant difference in PR, QRS, QTc and RR intervals (data not shown). Two PES protocols, S1S2 and S1S1 pacing, were introduced to the atrial septum to stress the hearts. *B*, PMCA1^cko^ hearts exhibited frequent short AT/AF when PES pacing was applied while none of the PMCA1^loxP/loxP^ hearts exhibited any arrhythmic events in ECG (left panel) and MAP (right panel) recordings. *C*, representative traces of ECG (upper panel) and MAP (lower panel) recordings under S1S1 PES pacing. PMCA1^cko^ hearts showed sustained or non‐sustained AT/AF when PES pacing was applied while none of the PMCA1^loxP/loxP^ hearts exhibited any arrhythmic events. *D*, the percentage occurrence of arrhythmic events as a result of different S1S1 PES pacing frequencies in PMCA1^cko^ and PMCA1^loxP/loxP^ hearts. *E*, the action potential duration at 90% (APD_90_) and effective refractory period (ERP) under baseline and series of S1S1 PES pacing (90, 70, 50 and 30 ms) showed no significant differences in PMCA1^loxP/loxP^ and PMCA1^cko^ mice under 1 mm [Ca^2+^]_o_ condition.

**Figure 3:**
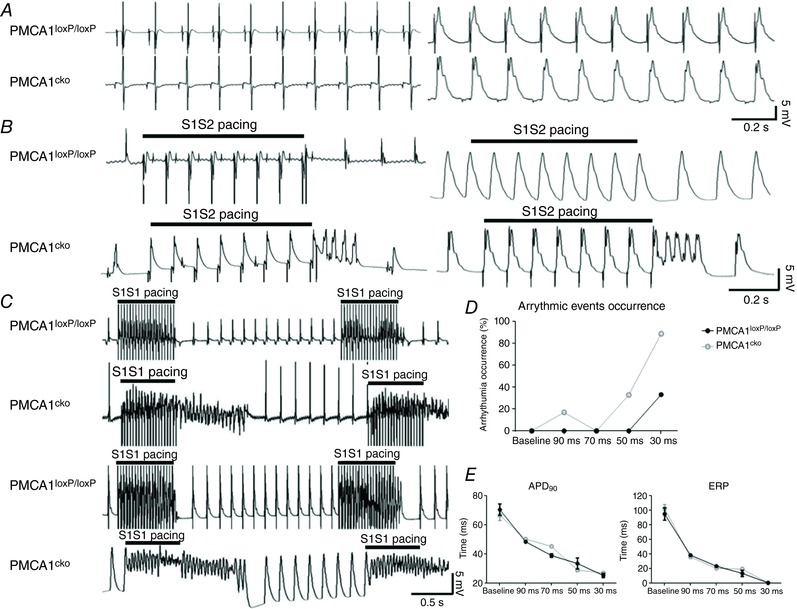
The *ex vivo* Langendorff recordings of ECG and MAP of PMCA1^cko^ and PMCA1^loxP/loxP^ hearts at 2 mm [Ca^2+^]_o_ *A*, representative traces of the *ex vivo* Langendorff ECG (left) and MAP (right) recordings. There was no significant difference in PR, QRS, QTc and RR intervals (data not shown). *B*, PMCA1^cko^ hearts exhibit frequent short AT/AF when PES pacing was applied whilst none of the PMCA1^loxP/loxP^ hearts exhibited any arrhythmic events of ECG (left panel) and MAP (right panel) recordings. *C*, representative traces of ECG (upper panel) and MAP (lower panel) recordings under S1S1 PES pacing. PMCA1^cko^ hearts showed sustained or non‐sustained AT/AF when PES pacing was applied while none of the PMCA1^loxP/loxP^ hearts exhibited any arrhythmic events. *D*, the percentage occurrence of arrhythmic events as a result of different S1S1 PES pacing frequencies in PMCA1^cko^ and PMCA1^loxP/loxP^ hearts. *E*, the APD_90_ and ERP under baseline and series of S1S1 PES pacing (90, 70, 50 and 30 ms) showed no significant differences in PMCA1^loxP/loxP^ and PMCA1^cko^ mice under 2 mm [Ca^2+^]_o_ conditions.

**Figure 4:**
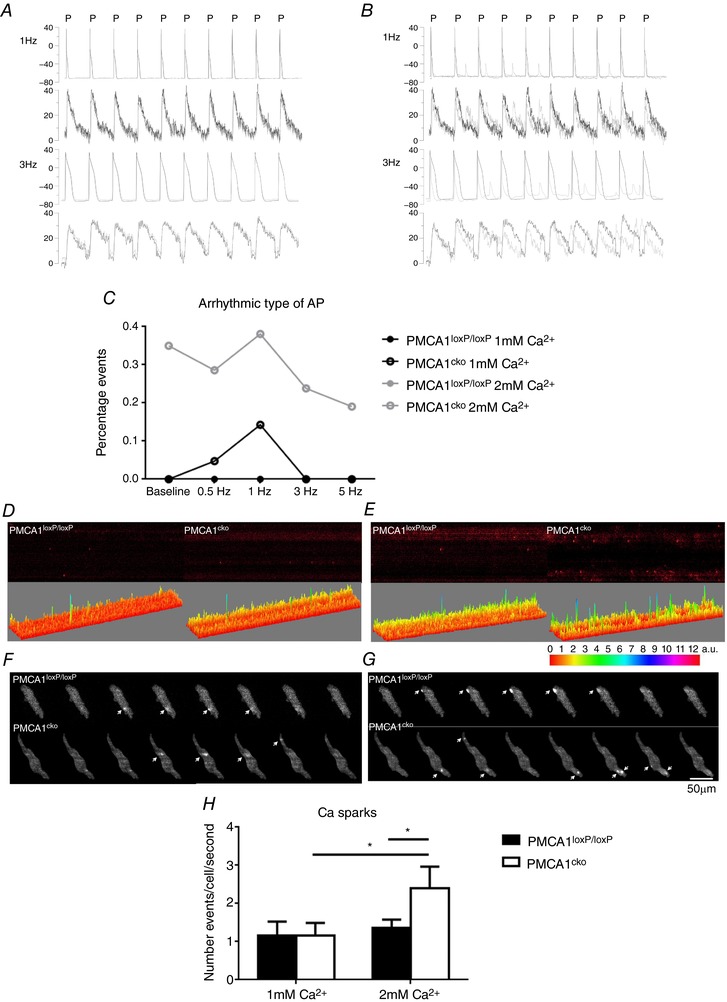
The occurrences of arrhythmic type of AP and calcium sparks in isolated PMCA1^cko^ and PMCA1^loxP/loxP^ atrial cells at 1 mm [Ca^2+^]_o_ and 2 mm [Ca^2+^]_o_ *A* and *B*, the characteristics of AP and corresponding calcium transient were analysed for PMCA1^cko^ (grey trace) mice and PMCA1^loxP/loxP^ controls (black trace) at 1 mm [Ca^2+^]_o_ (*A*) or 2 mm [Ca^2+^]_o_ (*B*) with 1 and 3 Hz stimulation. P indicates the stimulation pulse. *C*, there was an increase in arrhythmic type of AP occurrence in PMCA1^cko^ mice with faster stimulation frequency under 2 mm [Ca^2+^]_o_. *D* and *E*, the number of calcium sparks measured by linescan was higher in PMCA1^cko^ myocytes under both baseline (*D*) and 2 mm [Ca^2+^]_o_ (*E*) conditions. *F* and *G*, this increase in the number of calcium sparks was also demonstrated in PMCA1^cko^ myocytes measured with a spinning‐disc microscope under both baseline (*F*) and 2 mm [Ca^2+^]_o_ (*G*) conditions. *H*, the quantification of the number of calcium sparks per cell per second.

**Figure 5:**
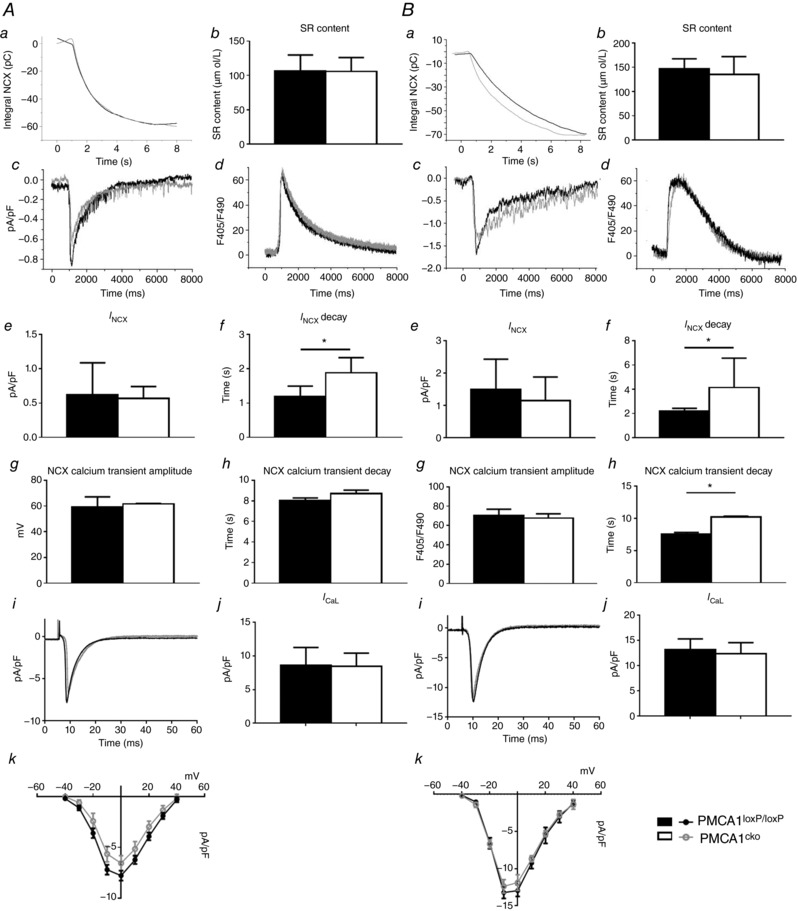
Calcium extrusion via NCX and SR content were examined in PMCA1^cko^ mice and PMCA1^loxP/loxP^ controls at 1 mm [Ca^2+^]_o_ (*A*) or 2 mm [Ca^2+^]_o_ (*B*) conditions Representative traces of integral NCX, caffeine‐induced current and corresponding calcium transient are shown under baseline (*Aa*, *c* and *d*) and 2 mm [Ca^2+^]_o_ (*Ba*, *c* and *d*) conditions. The time decay of *I*_NCX_ in PMCA1^cko^ myocytes was significantly prolonged under both baseline (*Af*) and 2 mm [Ca^2+^]_o_ (*Bf*) conditions. The time decay of corresponding calcium transient in PMCA1^cko^ myocytes showed significantly longer duration than PMCA1^loxP/loxP^ myocytes under 2 mm [Ca^2+^]_o_ (*Bh*) but not under baseline (*Ah*) conditions. There was no significant difference between the SR content of PMCA1^loxP/loxP^ and PMCA1^cko^ mice under baseline (*Ab*) and 2 mm [Ca^2+^]_o_ (*Bb*) conditions. The density of *I*_NCX_ and corresponding calcium transient amplitude showed no significant difference between the SR content of PMCA1^loxP/loxP^ and PMCA1^cko^ mice under baseline (*Ae* and *g*) and 2 mm [Ca^2+^]_o_ (*Be* and *g*) conditions. Representative traces of *I*_LTCC_ are shown under baseline (*Ai*) and 2 mm [Ca^2+^]_o_ (*Bi*) conditions. The peak amplitude of *I*_LTCC_ in PMCA1^cko^ myocytes and PMCA1^loxP/loxP^ myocytes at −10 mV were not significantly different under baseline (*Aj*) and 2 mm [Ca^2+^]_o_ (*Bj*) conditions, and the *I–V* curves of *I*_LTCC_ in PMCA1^cko^ myocytes and PMCA1^loxP/loxP^ myocytes were not significantly different under baseline (*Ak*) and 2 mm [Ca^2+^]_o_ (*Bk*) conditions.

In the analysis of the continuous experimental data (Figs 1 *B* and *H*, and 3–8), values are reported as means ± SEM of *n* experiments, and the observations were thought to be sufficiently close to a ‘normal’ distribution, as judged by Kolmogorov–Smirnov normality, to permit the use of parametric tests. Student's paired or unpaired *t* test was used as appropriate. Comparisons of more than one factor or between multiple groups utilized two‐way ANOVA, corrected by a Bonferonni *post hoc t* test if necessary. The analysis of categorical data (Figs 1 *D* and *G*, and 2 *B*) employed Fisher's exact test. In the statistical tests significance was taken to correspond to a probability ≤0.05. All *P*‐values are two‐sided, AND the statistical software used to generate the results was SPSS (SPSS Software, IBM Corp., Armonk, NY, USA) and Prism (GraphPad Software, La Jolla, CA, USA) software.

## Results

### Confirmation of the deletion of PMCA1 protein in atrial tissue in PMCA1^cko^ mice

The expression of PMCA1 protein in the atria of PMCA1^loxP/loxP^ and PMCA1^cko^ mice was analysed by western blotting and immunohistochemistry staining, respectively. As shown in Fig. 1 *A* and *B*, the expression of PMCA1 in the atria at the protein level was efficiently abolished in PMCA1^cko^ mice, while there is a clear expression of a 135 kDa PMCA1 protein in the PMCA1^loxP/loxP^ control littermates. The efficiency of cardiomyocyte‐specific knockout of PMCA1 in the atrial tissue section was further confirmed by immunohistochemistry staining as shown in Fig. 1 *C*. There was a strong intensity of PMCA1 labelling in the PMCA1^loxP/loxP^ atria tissue sections while the intensity was very low in PMCA1^cko^ atria. The mRNA levels of PMCA4 in both atria and ventricle were unaltered in PMCA1^loxP/loxP^ and PMCA1^cko^ mice as shown in Fig. 1 *D*. The *in vivo* HR of PMCA1^loxP/loxP^ and PMCA1^cko^ mice showed no significant difference with either 1‐week isoproterenol treatment or acute dobutamine injection treatment as shown in Fig. 1 *E*.

### PMCA1 deficiency increases susceptibility to atrial tachyarrhythmia in Langendorff‐perfused hearts under stress conditions

For the ECG characteristics, atrial MAPs were recorded in Langendorff perfused PMCA1^loxP/loxP^ (*n* = 7) and PMCA1^cko^ hearts (*n* = 8). The role of PMCA1 in maintaining atrial electrical stability was then investigated under baseline conditions (1 mm [Ca^2+^]_o_ Krebs solution); when subjected to Ca^2+^ overload produced by increasing the extracellular Ca^2+^ concentration ([Ca^2+^]_o_) to 2 mm [Ca^2+^]_o_ in the Krebs solution; and when hearts were subjected to PES.

Under basal conditions of sinus rhythm, there was no significant difference in *ex vivo* ECG and MAP characteristics between PMCA1^loxP/loxP^ and PMCA1^cko^ hearts (Fig. 2 *A*). However, when the hearts were subject to PES, either S1S1 burst pacing and S1S2 extra‐stimulus pacing, PMCA1^cko^ but not PMCA1^loxP/loxP^ had high incidences of atrial tachyarrhythmia including AT and AF (Fig. 2 *B* and *C*). The occurrence of AT/AF was particularly high following S1S1 burst pacing frequencies (basal cycle length (BCL) <50 ms) (Fig. 2 *D*). Only 1 out of 7 PMCA1^loxP/loxP^ hearts exhibited a short AF at 30 ms pacing interval accounting for only 0.8% of the total pacing duration under the S1S1 burst pacing protocol at 30 ms BCL, whereas short AT/AF were triggered in 8 out 8 PMCA1^cko^ hearts by S1S1 burst pacing, accounting for 11.8% of the total pacing duration.

However, when perfused with 2 mm [Ca^2+^]_o_ Krebs solution under sinus rhythm, ectopic beats and AP alternans were noticed in 1 out of 6 PMCA1^cko^ hearts. The PMCA1^loxP/loxP^ hearts remained under normal heart rhythm during and after both S1S2 and S1S1 pacing protocols. However, frequent, short episodes of AT were induced by S1S2 protocol pacing in PMCA1^cko^ hearts, and much longer durations of AT were induced by S1S1 pacing, particularly at higher S1S1 burst pacing frequencies (BCL < 50 ms). These short episodes of AF or AT were triggered by S1S2 pacing in 5 out of 6 PMCA1^cko^ hearts but none were recorded in PMCA1^loxP/loxP^ hearts. S1S1 burst pacing at high frequencies triggered severe and longer AT or AF in all PMCA1^cko^ hearts (Fig. 3 *B* and *C*). It was clear that most of the arrhythmic events were evoked at high frequencies (cycle length ∼30–50 ms) in PMCA1^cko^ hearts, and that more severe AT/AF was seen in PMCA1^cko^ hearts under 2 mm [Ca^2+^]_o_ Krebs solution (Fig. 3 *D*) than under baseline conditions.

### PMCA1 deficiency produces instabilities in membrane potentials and Ca^2+^ handling in atrial myocytes

Isolated PMCA1^loxP/loxP^ and PMCA1^cko^ atrial myocytes demonstrated significant differences in Ca^2+^ homeostasis and stability in membrane potentials. Figure 4 *A* and *B* compares APs and Ca^2+^ transients from PMCA1^loxP/loxP^ and PMCA1^cko^ myocytes following stimulation at 1 and 3 Hz field and perfusion with 1 mm [Ca^2+^]_o_ (Fig. 4 *A*) or 2 mm [Ca^2+^]_o_ (Fig. 4 *B*) Tyrode solutions. PMCA1^cko^ myocytes showed higher occurrences of arrhythmia‐like APs and Ca^2+^ events (delayed after‐depolarization (DADs) and irregular, alternating Ca^2+^ transients and/or Ca^2+^ waves) with increased stimulation frequencies than PMCA1^loxP/loxP^ myocytes (Fig. 4 *C*).

These instabilities correlated with differences in evoked Ca^2+^ sparks in the cytosol as measured by linescan. Thus increased Ca^2+^ sparks in PMCA1^cko^ myocytes occurred under both baseline (Fig. 4 *D* right) and 2 mm [Ca^2+^]_o_ (Fig. 4 *E* right) conditions compared to PMCA1^loxP/loxP^ myocytes under both baseline (Fig. 4 *D* left) and 2 mm [Ca^2+^]_o_ (Fig. 4 *E* left) conditions. The increased number of Ca^2+^ sparks was also demonstrated in PMCA1^cko^ myocytes measured by spinning‐disc microscopy compared to PMCA1^loxP/loxP^ myocytes under both baseline (Fig. 4 *F*, bottom) and 2 mm [Ca^2^]_o_ (Fig. 4 *G*, bottom) conditions. Under 1 mm [Ca^2+^]_o_ conditions, the number of Ca^2+^ sparks per second in PMCA1^loxP/loxP^ atrial myocytes (*n* = 5) was 1.17 ± 0.16 s^−1^, which was similar to that seen in PMCA1^cko^ atrial myocytes (1.16 ± 0.10 s^−1^) (*n* = 11). In PMCA1^cko^ atrial myocytes (*n* = 11), but not PMCA1^loxP/loxP^ atrial myocytes (1.35 ± 0.10 s^−1^, *n* = 4), the number of Ca^2+^ sparks was significantly increased to 2.40 ± 0.17 s^−1^ under 2 mm [Ca^2+^]_o_ (Fig. 4 *H*).

These instabilities correlated with differences in evoked cytosolic and SR Ca^2+^ responses and NCX (Fig. 5) among PMCA1^loxP/loxP^ (black traces) and PMCA1^cko^ myocytes (grey traces) under either baseline (Fig. 5 *A*) or 2 mm [Ca^2^]_o_ (Fig. 5 *B*).

(i) Peak systolic [Ca^2+^]_i_ was estimated from the differences between peaks and baselines of systolic Ca^2+^ transients obtained during regular stimulations. This was indistinguishable between PMCA1^loxP/loxP^ myocytes (*n* = 26) and PMCA1^cko^ myocytes (*n* = 25) under either baseline or 2 mm [Ca^2^]_o_ conditions.
(ii) SR Ca^2+^content measured by integration of *I*_NCX_ was indistinguishable between PMCA1^loxP/loxP^ (*n* = 7) and PMCA1^cko^ (*n* = 15) myocytes under either baseline [Ca^2^]_o_ or 2 mm [Ca^2^]_o_ conditions (Fig. 5 *Aa*, *b* and *Ba*, *b*, respectively).
(iii) Decay of caffeine‐induced Ca^2+^ transients provided estimates of rates of Ca^2+^ removal by NCX from the cytosol. The time decay of *I*_NCX_ in PMCA1^cko^ myocytes (*n* = 7) was prolonged significantly compared to PMCA1^loxP/loxP^ myocytes (*n* = 15) under both baseline and 2 mm [Ca^2^]_o_ conditions (Fig. 5 *Af* and *Bf*, respectively).
(iv) The density of *I*_NCX_ and corresponding Ca^2+^ transient amplitude showed no significant difference between PMCA1^loxP/loxP^ and PMCA1^cko^ myocytes under baseline (Fig. 5 *Ae–h*) and 2 mm [Ca^2^]_o_ (Fig. 5 *Be–h*) conditions.
(v) Finally the *I*_LTCC_ was measured under voltage clamp of PMCA1^loxP/loxP^ myocytes (*n* = 23) and PMCA1^cko^ myocytes (*n* = 19). There was no significant difference in the density of *I*_LTCC_ between PMCA1^loxP/loxP^ myocytes (8.7 ± 0.49 pA pF^−1^) and PMCA1^cko^ myocytes (6.13 ± 0.77 pA pF^−1^) (Fig. 5 *Ai–k* and *Bi–k*).

Together these findings demonstrate that PMCA1 deficiency alters cellular Ca^2+^ homeostasis under baseline conditions and when subjected to Ca^2+^ overload (2 mm [Ca^2^]_o_) stress.

## Discussion

Whilst it is evident from several recent genome‐wide association studies that PMCA1 has a strong association with hypertension and blood pressure variance in humans (Benkwitz *et al*. 1999; Gros *et al*. 2003), its physiological role in the atria is not known. Thus, we have begun to unravel the involvement of PMCA1 in atrial electrophysiology and Ca^2+^ homeostasis by studying a cardiomyocyte‐specific deletion of PMCA1 in a mouse model.

It is generally considered that PMCAs only make a minor contribution to Ca^2+^ efflux compared with NCX (Bers, 2000, 2002); however, several lines of evidence from this study indicate that PMCA1 is crucial for maintaining atrial Ca^2+^ homeostasis and electrical stability under stress conditions although its role in Ca^2+^ extrusion under baseline physiological conditions is likely not significant.

At the whole heart level, under 1 mm [Ca^2+^]_o_ Kreb solution basal conditions without pacing, there was no significant difference between PMCA1^loxP/loxP^ and PMCA1^cko^ *ex vivo* ECG characteristics such as PR interval, QRS interval, PP interval and HR, indicating that the deletion of PMCA1 does not affect intracellular Ca^2+^ homeostasis and handling under physiological conditions. It has been reported that PMCA1 pumps only serve about 1% of Ca^2+^ extrusion in rabbit ventricular myocytes (Ferrier & Howlett, 2001; Bers, 2002), such a low contribution suggests that with the absence of the PMCA1 pump, NCX is able to compensate for the amount of Ca^2+^ efflux which should be pumped out by the PMCA1 pump. However, with pacing, particularly at high frequency burst pacing with an S1S1 protocol, frequent AT or AF was induced in PMCA1^cko^ hearts, while the PMCA1^loxP/loxP^ hearts remained in normal heart rhythm. The higher the pacing frequency, the more severe the atrial arrhythmias in PMCA1^cko^ hearts. This indicates that the hearts without PMCA1 could not maintain normal Ca^2+^ cycling under moderate or severe stress conditions such as burst pacing by S1S1 pacing that has been used for determining atrial repolarization alternans threshold. Deletion of PMCA1 may lead to an accumulation of diastolic cytosolic Ca^2+^ ions in the cell under stress conditions, which may lead to the occurrence of DAD. In PMCA1^cko^ hearts under pacing conditions, particularly at high frequencies, the NCX has to work with not only high capacity but also high affinity to keep up with the pacing frequency as well as covering the additional work of the PMCA1 pump. PMCA1 is more important for local organization of Ca^2+^ signalling (Penniston & Enyedi, 1998; Shaul & Anderson, 1998; Schuh *et al*. 2001) with low transport capacity (Carafoli, 1991b) and high affinity of Ca^2+^ and 1:1 Ca^2+^/ATP stoichiometry (Carafoli, 1991a). On the other hand, NCX is a low affinity with high capacity channel for Ca^2+^ ion transport (Brini & Carafoli, 2011). The natural characteristics of NCX and PMCA suggest that NCX could compensate for the deletion of PMCA1 under physiological conditions without pacing, but the PMCA1 pumps appear more important under stress conditions demanding fast Ca^2+^ removal.

Under 1 mm [Ca^2+^]_o_ baseline conditions with PES, the occurrences of atrial arrhythmias in PMCA1^cko^ hearts were generally more frequent than in PMCA1^loxP/loxP^ hearts with MAP recordings (Fig. 2). The occurrence of atrial arrhythmias under S1S1 pacing was higher than under S1S2 pacing, particularly with high frequency of pacing in PMCA1^cko^ hearts under physiological conditions. No significant difference was observed in action potential duration at 90% (APD_90_) and effective refractory period (ERP) between PMCA1^loxP/loxP^ and PMCA1^cko^ hearts. Bayer *et al*. studied the changes in amplitude and duration of ion currents against pacing frequency with a dynamic pacing protocol, and showed that 88–94% of the current amplitude in LTCCs was reduced at high pacing frequency compared to low pacing frequency (Bayer *et al*. 2010). As the Ca^2+^ buffering power is homeostatically regulated, the amount of Ca^2+^ influx via LTCCs should be equivalent to the amount of Ca^2+^ efflux through NCX and PMCA (Eisner *et al*. 2000). With increased pacing frequency the amplitude of *I*_LTCC_ was reduced, thereby leading to a reduction in Ca^2+^ efflux. However, the accelerated speed of Ca^2+^ efflux was required. Because NCX cannot facilitate high speed Ca^2+^ efflux, intracellular Ca^2+^ homeostasis was disturbed with increased [Ca^2+^]_i_ leading to atrial arrhythmias in PMCA1^cko^ hearts. Hence PMCA1 contributes a minor role in physiological conditions, but it plays an important role in intracellular Ca^2+^ homeostasis with increased pacing frequency.

When 2 mm [Ca^2+^]_o_ was applied to induce Ca^2+^ overload conditions without or with PES, the ECG parameters such as PR, QRS, PP intervals and HR, were not significantly different between PMCA1^loxP/loxP^ and PMCA1^cko^ hearts. The MAP recordings under such Ca^2+^ overload conditions, without pacing, indicated the appearance of DAD‐type APs in PMCA1^cko^ hearts (Fig. 3). This may be due to increased [Ca^2+^]_i_, with the extra Ca^2+^ ions binding to RyR and causing diastolic Ca^2+^ release during ERP. The mechanism of ectopic activities of the heart appears to depend on the amount of [Ca^2+^]_i_ (Hirose & Laurita, 2007). It is known that an increase in [Ca^2+^]_i_ can lead to DAD and trigger arrhythmias in atrial and ventricular cardiac myocytes (Song *et al*. 1992). Under 2 mm [Ca^2+^]_o_ conditions with S1S2 and S1S1 pacing, all of the PMCA1^loxP/loxP^ hearts remained in rhythmic AP while all of the PMCA1^cko^ hearts exhibited variable durations and severities of arrhythmias. The occurrence of atrial arrhythmias under S1S1 pacing was higher than that under S1S2 pacing, particularly with high frequency of pacing in PMCA1^cko^ hearts as shown in Fig. 4. Under both pacing and Ca^2+^ overload conditions, PMCA1^cko^ hearts showed more severe arrhythmias as the Ca^2+^ handling homeostasis became less balanced.

According to the MAP results with S1S1 pacing (Figs 3 and 4), the phenotypes of the frequency‐dependent arrhythmia (including AT and AF) appeared more often under high pacing frequencies (∼50–30 ms) in PMCA1^cko^ mice subjected to 1 and 2 mm [Ca^2+^]_o_ conditions. Many studies show high pacing frequency is associated with an increased Ca^2+^ transient and cardiac contraction, which implies that a Ca^2+^‐dependent mechanism is involved (Attwell *et al*. 1981; Joulin *et al*. 2009). The rate of contraction and relaxation is a fundamental characteristic of cardiomyocytes, and the rate of contraction and relaxation increases with increased pacing frequency (Masao, 2004). The occurrence of arrhythmias under 2 mm [Ca^2+^]_o_ in PMCA1^cko^ hearts was more often than under 1 mm [Ca^2+^]_o_ conditions. With the absence of PMCA1, the degree of the disruption to the Ca^2+^ cycling became more obvious with increased pacing frequencies under both 1 and 2 mm [Ca^2+^]_o_ conditions. In parallel, the frequency‐dependent pacing also disrupts the Ca^2+^ handling via changes in amplitude of current, kinetics and inactivation rate of LTCCs (Tiaho *et al*. 1994; Barrère‐Lemaire *et al*. 2000). Under stress conditions, the contribution of PMCA1 to Ca^2+^ handling and intracellular Ca^2+^ regulation therefore appears more important.

Under 1 mm [Ca^2+^]_o_ steady state conditions the characteristics of atrial myocyte APs, *I*_LTCC_ and corresponding Ca^2+^ transients are not altered by deletion of PMCA1 and the function of NCX and SR Ca^2+^ content, when examined by application of 10 mm caffeine, remained unaffected. However, the rate of decay of the NCX current in PMCA1^cko^ myocytes was significantly slower than that of PMCA1^loxP/loxP^ myocytes. The results indicate that PMCA1 plays only a minor role in Ca^2+^ cycling in atrial myocytes under physiological conditions.

Our results also indicate that LTCC current in PMCA1^cko^ myocytes was unaffected (the density of LTCC current in PMCA1^cko^ myocytes is not statistically different compared to that in PMCA1^loxP/loxP^ myocytes), suggests that Ca^2+^ influx to trigger the SR Ca^2+^ release was similar in PMCA1^loxP/loxP^ and PMCA1^cko^ myocytes. To maintain Ca^2+^ homeostasis in the myocyte, the amount of Ca^2+^ that enters the myocyte via LTCCs is approximately equivalent to the amount of Ca^2+^ to be removed by NCX and PMCA (Eisner *et al*. 2000).

With 10 mm caffeine application for 45 s to the atrial myocytes, the SR Ca^2+^ store was emptied and therefore the integrated NCX current indicated the SR content (Negretti *et al*. 1993). From our results, the NCX current amplitude in PMCA1^cko^ myocytes was similar compared with in PMCA1^loxP/loxP^ myocytes, but the decay time of the NCX current was significantly prolonged. As PMCA1 pumps have been deleted, NCX works alone to extrude Ca^2+^ and can still fully compensate for the loss of PMCA1 pumps.

However, as shown in Fig. 5, when the [Ca^2+^]_o_ was increased from 1 to 2 mm, two major phenomena were observed. Firstly, there was a potentially significant prolongation in the rate of NCX current decay in PMCA1^cko^ myocytes, which suggests that a higher [Ca^2+^]_o_ produces Ca^2+^ overload for the myocytes, and therefore NCX has to work harder and longer to compensate for the loss of the PMCA1 pumps in these cells. Such results are consistent with previous findings by Eisner's group (Diaz *et al*. 1997). They examined the effects of [Ca^2+^]_o_ on the [Ca^2+^]_i_, SR contents and NCX currents in rat ventricular myocytes, and found that a rise in [Ca^2+^]_o_ led to a decrease in the rate of Ca^2+^ removal from the cytosol as measured from the rate of decay of the caffeine‐induced response. Secondly, there was an increase in the number of spontaneous DAD‐type APs with corresponding Ca^2+^ transients in PMCA1^cko^ myocytes without stimulation. Such results indicated that increased Ca^2+^ overload due to a rise in [Ca^2+^]_o_ leads to increased SR content, triggering spontaneous SR Ca^2+^ release, and causing early after‐depolarization (EAD)‐ or DAD‐type and spontaneous APs. Our findings are thus consistent with the results from Eisner's groups, as they found that increased [Ca^2+^]_o_ leads to increased frequency of spontaneous Ca^2+^ release associated with increased SR content, and more frequent oscillation of the myocytes.

Another stress condition applied to the myocytes in this study was frequency‐dependent stimulation. As the results show in Fig. 4, the APs were rhythmic and synchronous with the stimulation at all frequencies (0.5, 1 and 3 Hz) in PMCA1^loxP/loxP^ myocytes; however, in PMCA1^cko^ myocytes, frequent EAD‐ or DAD‐type APs with corresponding spontaneous Ca^2+^ transients occurred, particularly when Ca^2+^ overload conditions were combined with high frequencies (3 and 5 Hz). These results coincide with the *ex vivo* heart studies (Figs 2 and 3), as more atrial arrhythmias occurred at a high frequency under Ca^2+^ overload. As discussed above, the NCX could not compensate for the workload of the PMCA1 pump in PMCA1^cko^ myocytes even with prolonged decay rates. We also found this overload of [Ca^2+^]_i_ was severe in some myocytes. As PMCA1 pumps have high affinity in Ca^2+^ transport with fast extrusion, under stress conditions, they can remove Ca^2+^ faster and more efficiently than NCX. While as NCX is a housekeeping exchanger for Ca^2+^ extrusion, it could compensate for the deletion of PMCA1 pumps under physiological conditions, but not under stress conditions.

Our results indicate an important role for PMCA1 in atrial myocyte Ca^2+^ handling under physiological stress conditions that has not been previously reported, which may lead us to rethink its role in the heart. In contrast to ventricular myocytes, the role of t‐tubules in atrial myocytes is less clear. For a long time, the canonical atrial myocyte was characterized by sparse transverse tubule invaginations and slow intracellular Ca^2+^ propagation. However, a recent study by Brandenburg *et al*. (2016) has identified a membrane structure and Ca^2+^‐signalling complex that may enhance the speed of atrial contraction independently of phospholamban regulation. This axial couplon was observed in human and mouse atria and is composed of voluminous axial tubules with extensive junctions to the SR that include ryanodine receptor 2 (RyR2) clusters. They found that in mouse atrial myocytes, axial tubule structures triggered Ca^2+^ release from the SR approximately two times faster at the centre of the atrial myocyte than at the surface. Rapid Ca^2+^ release correlated with colocalization of highly phosphorylated RyR2 clusters at axial tubule–SR junctions and earlier, more rapid shortening of central sarcomeres. This may suggest different local control of excitation–contraction coupling in atrial myocytes compared to ventricular myocytes. The development of local control theories in cardiac excitation–contraction coupling solved a major problem in the Ca^2+^‐induced Ca^2+^ release hypothesis. Local control explained how regeneration, inherent in the Ca^2+^‐induced Ca^2+^ release mechanism, might be limited spatially to enable graded Ca^2+^ release (and force production). The key lies in the stochastic recruitment of individual Ca^2+^ release units (or couplons) where adjacent Ca^2+^ release units are partially uncoupled by the distance between them. In the Ca^2+^ release unit, individual groups of sarcoplasmic reticulum Ca^2+^ release channels (RyRs) are very close to the surface membrane where Ca^2+^ influx, controlled by membrane depolarization, leads to high local Ca^2+^ levels that enable a high speed response from RyRs that have a very low probability of opening at resting Ca^2+^ levels (Cannell & Kong, 2012).

In conclusion, our study shows that as a fast Ca^2+^ remover, the PMCA1 pump has a minor role under baseline physiological conditions, and as a slow steady Ca^2+^ exchanger, NCX can compensate for the workload of the PMCA1 pump. However, under stress conditions (Ca^2+^ overload or frequency‐dependent stimulation conditions or both) PMCA1 becomes more important not only for its contribution in Ca^2+^ extrusion but also for its role in eletrophysiological stability, and is thus critical for maintaining atrial Ca^2+^ homeostasis. This is particularly critical during fast removal of Ca^2+^ from the cytosol which is required under stress conditions.

### Limitations of the study

In this study, the aspect of the role of PMCA1 in subcellular Ca^2+^ events, such as its role in regional spontaneous Ca^2+^ spark activity, was not investigated. It will be an interesting area to examine in the future. Previous studies indicated that cells lacking t‐tubules such as atrial myocytes and neonatal myocytes show variable spontaneous Ca^2+^ spark activity (Sheehan *et al*. 2006). In neonatal cardiac myocytes, where the t‐tubular system is still developing, Ca^2+^ sparks are restricted to the cell periphery and are associated with caveolae (Löhn *et al*. 2000), while in rabbit Purkinje cells, which also lack t‐tubules but contain peripheral and central RyRs (Cordeiro *et al*. 2001), Ca^2+^ sparks occur only at the cell periphery, even during β‐adrenergic stimulation. However, in contrast, in canine Purkinje cells, Ca^2+^ sparks occur ubiquitously throughout the cell (Stuyvers *et al*. 2005), while in rat atrial cells, Ca^2+^ sparks have been observed in the central and subsarcolemmal regions of the myocytes with no significant differences in frequency, amplitude or kinetics (Stuyvers *et al*. 2005). Furthermore, aside from different frequencies, peripheral and central Ca^2+^ sparks in rat atrial myocytes have also been found to have differing spatiotemporal characteristics (Woo *et al*. 2003), and it has been suggested that the higher frequency of peripheral Ca^2+^ sparks may result from interactions between the α_1C_ subunit of the dihydropyridine receptor with the RyR (Woo *et al*. 2003).

## Additional information

### Competing interests

The authors declare no competing financial interests.

### Author contributions

Y.W.: acquisition, analysis or interpretation of data for the work, drafting the work, manuscript writing; C.W.: acquisition, analysis or interpretation of data for the work; M.L.: conception and design of the work, revising it critically for important intellectual content, manuscript writing; E.C.: conception and design of the work, provision of study animals, revising it critically for important intellectual content. All authors have approved the final version of the manuscript and agree to be accountable for all aspects of the work. All persons designated as authors qualify for authorship, and all those who qualify for authorship are listed.

### Funding

This study was supported by MRC (G10002647, G1002082, M.L., E.C.), BHF (PG/14/80/31106, PG/16/67/32340, PG/12/21/29473, PG/11/59/29004 M.L., E.C.), BHF CRE at Oxford (M.L.) grants.
